# Genetic Structure of a Local Population of the *Anopheles gambiae* Complex in Burkina Faso

**DOI:** 10.1371/journal.pone.0145308

**Published:** 2016-01-05

**Authors:** Kyriacos Markianos, Emmanuel Bischoff, Christian Mitri, Wamdaogo M. Guelbeogo, Awa Gneme, Karin Eiglmeier, Inge Holm, N’Fale Sagnon, Kenneth D. Vernick, Michelle M. Riehle

**Affiliations:** 1 Program in Genomics, Boston Children's Hospital, Harvard Medical School, Boston, Massachusetts, 02115, United States of America; 2 Institut Pasteur, Unit of Insect Vector Genetics and Genomics, Department of Parasites and Insect Vectors, CNRS Unit of Hosts, Vectors and Pathogens (URA3012), Paris, 75015, France; 3 CNRS, Unit of Hosts, Vectors and Pathogens (URA3012), 28 rue du Docteur Roux, Paris, 75015, France; 4 Centre National de Recherche et de Formation sur le Paludisme, 01 BP 2208 Ouagadougou, Burkina Faso; 5 University of Minnesota, Department of Microbiology, Saint Paul, Minnesota, 55108, United States of America; National Institute for Communicable Diseases/NHLS, SOUTH AFRICA

## Abstract

Members of the *Anopheles gambiae* species complex are primary vectors of human malaria in Africa. Population heterogeneities for ecological and behavioral attributes expand and stabilize malaria transmission over space and time, and populations may change in response to vector control, urbanization and other factors. There is a need for approaches to comprehensively describe the structure and characteristics of a sympatric local mosquito population, because incomplete knowledge of vector population composition may hinder control efforts. To this end, we used a genome-wide custom SNP typing array to analyze a population collection from a single geographic region in West Africa. The combination of sample depth (n = 456) and marker density (n = 1536) unambiguously resolved population subgroups, which were also compared for their relative susceptibility to natural genotypes of *Plasmodium falciparum* malaria. The population subgroups display fluctuating patterns of differentiation or sharing across the genome. Analysis of linkage disequilibrium identified 19 new candidate genes for association with underlying population divergence between sister taxa, *A*. *coluzzii* (M-form) and *A*. *gambiae* (S-form).

## Introduction

Throughout sub-Saharan Africa, members of the *Anopheles gambiae* species complex are primary vectors of the human malaria parasite, *Plasmodium falciparum*, which is responsible for extensive human morbidity and mortality. Heterogeneity within the *A*. *gambiae* complex for ecological preference, feeding behavior, and *Plasmodium* susceptibility stabilize and expand the malaria vectorial system in nature [1, 2]. Phenotypic differences for these traits can vary between population subgroups or among individuals within a subgroup, and are influenced by genetic variation [3–9].

Previous studies have characterized population structure of the *A*. *gambiae* species complex, focusing on the ‘chromosomal forms’ carrying non-random combinations of segregating paracentric inversions [6–8], and also on the reproductively isolated subgroups originally named the M and S molecular forms [10–12]. The latter were recently renamed as *A*. *coluzzii* and *A*. *gambiae*, respectively, sister taxa within the *A*. *gambiae* species complex that also contains 6 additional species [13]. To date, most studies have examined population structure by genotype analysis of candidate loci using panels of microsatellite markers [11, 14, 15]. Genotyping using single-nucleotide polymorphism (SNP) array technology was first explored in a study of genomic regions that are differentiated between sympatric *A*. *coluzzii* and *A*. *gambiae*, termed speciation islands (SI) [16–18], although the role of these islands in population differentiation or speciation remains unresolved [19]. A custom SNP array, similar to the one used here but focusing mainly on candidate insecticide resistance loci, was used to screen large numbers of samples for novel insecticide resistance loci as well as for assessment of population subdivision [18, 20].

The distribution of *A*. *coluzzii* and *A*. *gambiae* across West Africa is correlated with ecological factors [21–23], and the two species display different frequencies of the *kdr* insecticide-resistance allele, a coding SNP (variant L1014F) of the *para* gene encoding a voltage-gated sodium ion channel [24]. Genetic analysis reveals additional levels of substructure within *A*. *coluzzii* [11] and *A*. *gambiae* populations [15], which has not yet been fully characterized. *A*. *coluzzii* and *A*. *gambiae* were initially thought to be highly reproductively isolated, but elevated rates of hybridization between them were described in certain geographic zones [25–27], and more recent work has shown that introgression between the two sister species is widespread and extensive [28] as is introgression with another closely related species, *A*. *arabiensis* [29].

Mosquito sampling strategies for studies of vector populations vary widely, from punctual collections representative of a particular geographic location at one time point (e.g. [30]) to repeated sampling of a site over time (e.g. [28]). Most population studies of the *A*. *gambiae* species complex have sampled broadly across geography but not deeply, that is, sample sizes per site tend to be relatively small [21, 31]. As our goal was comprehensive analysis of a local mosquito population, we generated large collections from a single local population in Burkina Faso over two transmission seasons. Initial analysis of this population using a limited number of microsatellite loci identified, in addition to *A*. *coluzzii* and *A*. *gambiae*, a novel subgroup named Goundry [14], an apparent founder population that may have originated by introgression between *A*. *coluzzii* and *A*. *gambiae* [28], followed by establishment of mating barriers between Goundry and the sympatric *A*. *coluzzii* and *A*. *gambiae*. The latter two species share extensive variation, cluster closely together [29], and display a deeper separation from the Goundry subgroup than from each other [14, 28].

Here, we designed a custom SNP array using the Illumina Golden Gate genotyping platform to analyze additional population samples and their metadata, including resting behavior and malaria susceptibility. Resting behavior is important because most vector control tools target indoor-resting mosquitoes [7]. Our goal was a comprehensive analysis of population structure within a deeply sampled local vector population. Given the higher density of SNP markers used as compared to a previous analysis of the same local population [14], we were also able to examine variation of differentiation patterns across the genome. Genotyping of a medium density SNP marker set (n = 1536) in a large number of individual samples occupies an efficient analytical niche that balances cost and effort. A high-density Affymetrix array [32] or whole genome sequencing provide more information per sample, but high per-sample cost limits the practical sample size and may diminish the attractiveness of these approaches for population-based studies. The feature density of the current study was more than sufficient for identification of population substructure. We present an approach to acquire genome-wide variation data from deep samples, while balancing cost and effort.

## Results

### Comprehensive detection of subdivision in a local population

Using a custom designed SNP chip we analyzed population subdivision in a deeply sampled local vector population in Burkina Faso. We first hybridized a pilot (n = 96) and then an expanded (n = 384) set of samples. The first 96 samples were used to validate array performance and included duplicates (n = 24) to verify reproducibility of genotype calls. The 72 unique samples in the pilot set included indoor-resting collections of *A*. *coluzzii* (n = 11), *A*. *gambiae* (n = 12), larval collections of Goundry (n = 19) as well as sibling species *A*. *arabiensis* (n = 30). Importantly, the expanded set of 384 samples were chosen based solely on their participation in a successful experimental feeding on malaria-infective blood and thus, aside from taking a bloodmeal, constituted an unbiased set of population samples.

**Fig 1:**
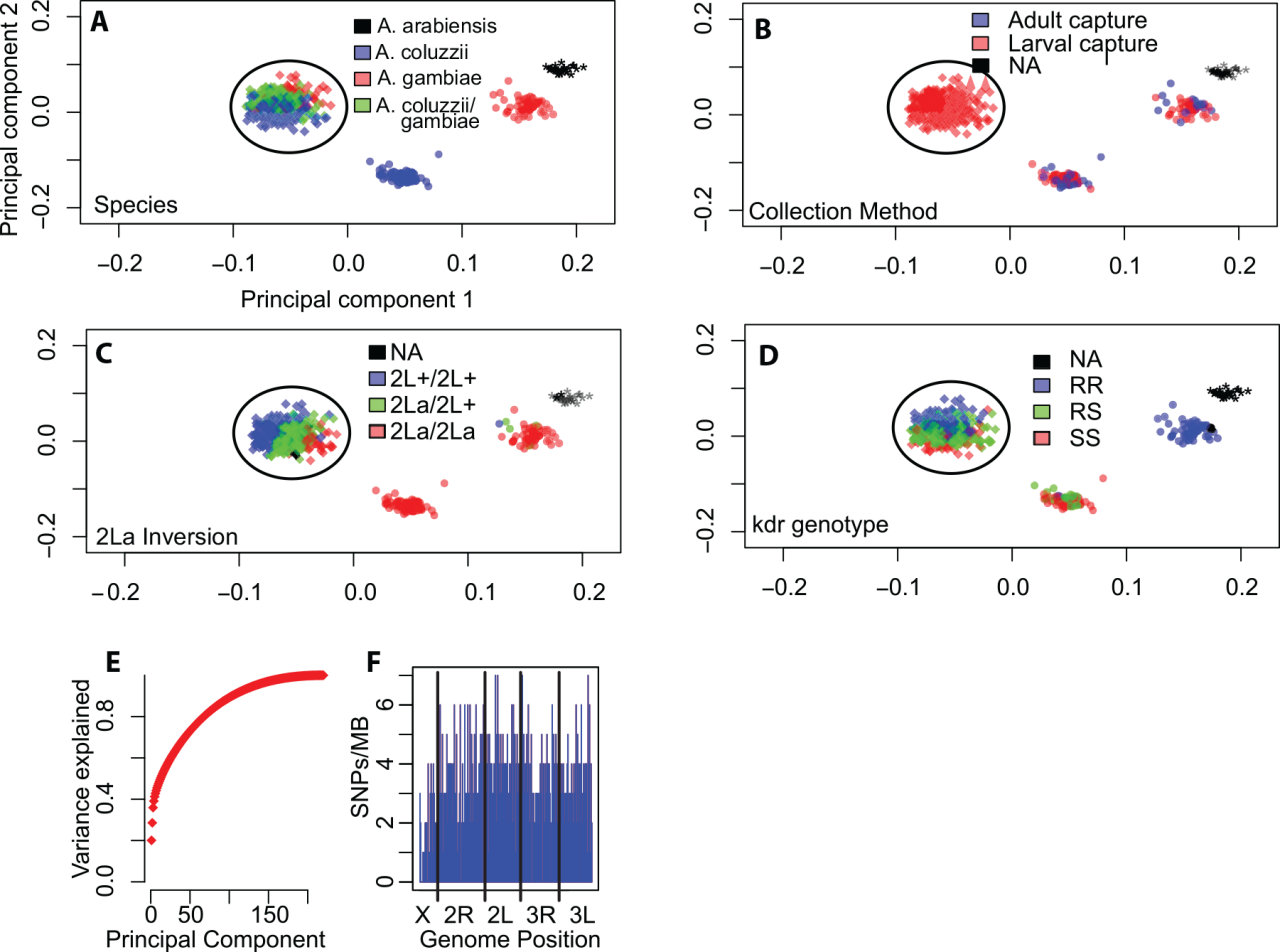
A comprehensive image of population structure is provided by genome-wide SNP typing in a local *Anopheles* population. Principal component analysis (PCA) was performed on 812 genome-wide, uniformly spaced SNPs typed in 422 individual mosquitoes collected in the village of Goundry, Burkina Faso over two years. A-F, Symbol color represents genetic attributes determined by molecular assays. A) species, B) collection method, C) genotype of 2La inversion, and D) genotype of kdr insecticide resistance-associated SNP. Axis labels for (A-D) as in (A). E) The cumulative variance of the PCA explained as a function of the number of principal components. The first two components explain greater than 25% of the variation. F) Distribution of SNP markers across the genome. Vertical blue bars indicate the number of SNPs per Mb, vertical black bars indicate the breakpoints between chromosome arms. The circled cluster in all panels indicates those individuals belonging to the Goundry form.

**Fig 2:**
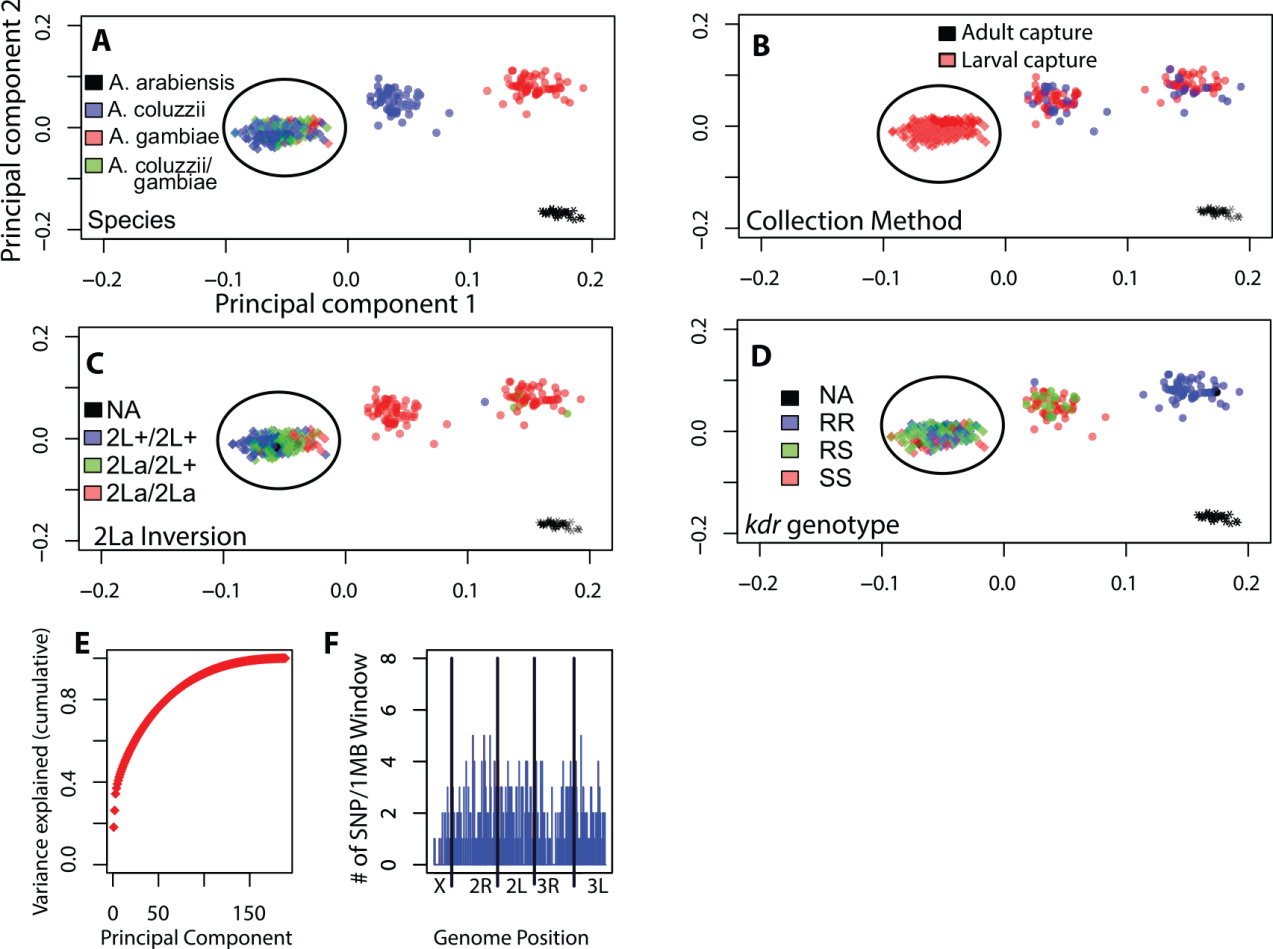
Identical population substructure is detected with half the number of SNPs. The same PCA analyses as in Fig 1 were repeated with 400 randomly sub-sampled SNPs, revealing the same four population subgroups. Panel labels (A-F) as in Fig 1. Note the Principal Component 2 is in opposite polarity to Fig 1, hence the presence of the *A*. *arabiensis* cluster in the lower right hand corner. Circled cluster indicates individuals belonging to the Goundry form.

**Fig 3:**
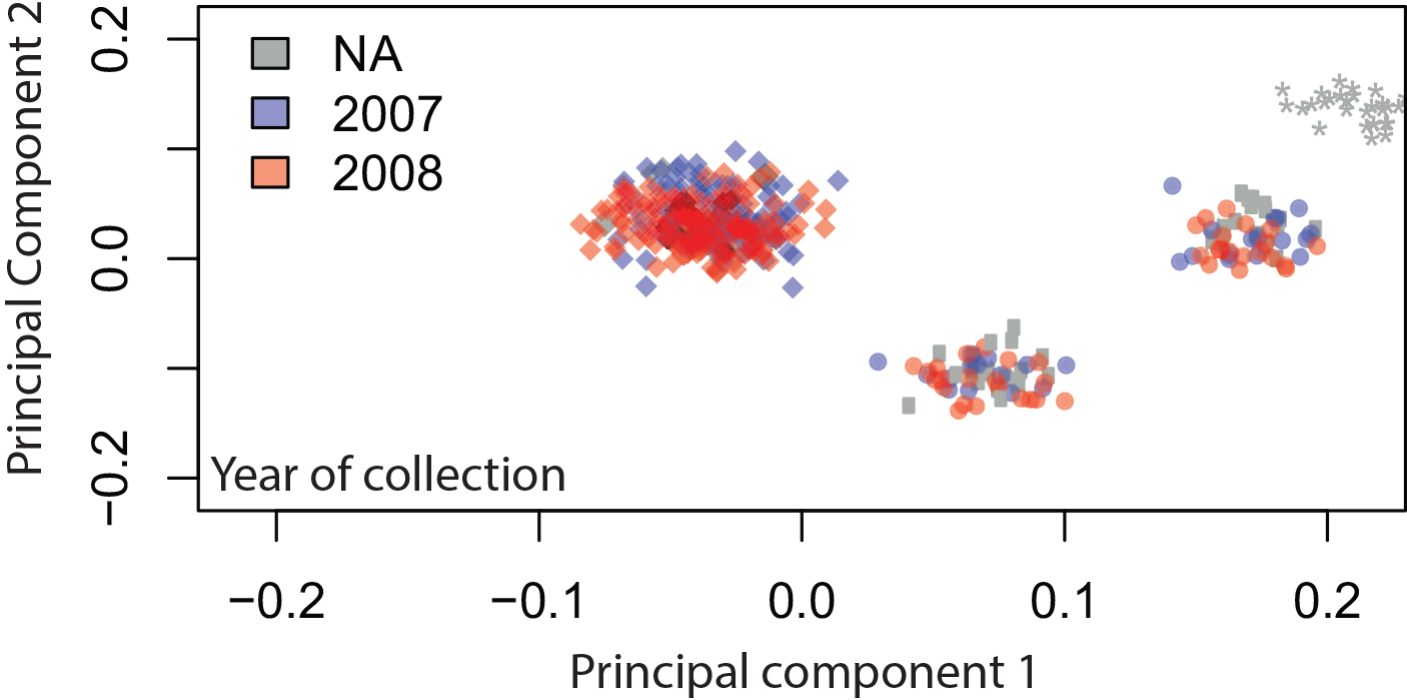
Population subdivision is comparable across two malaria transmission seasons. Samples are colored by collection year, 2007 (red) and 2008 (blue). The 96 pilot samples used for initial quality control are indicated in gray. There is no significant difference in the composition of the local mosquito population across years (chi-square = 0.457, df = 2, p = 0.796).

Genotypes generated by the uniformly-spaced genome-wide marker set revealed four distinct clusters when analyzed by principal component analysis (PCA). Overlay of species diagnostic results (Fig 1A) indicates the presence of *A*. *coluzzii*, *A*. *gambiae*, *A*. *arabiensis*, and a cluster where both *A*. *coluzzii* and *A*. *gambiae* species markers are present, the Goundry form, a discrete group with undetermined taxonomic status [14, 28]. Behavioral metadata (Fig 1B) indicate that the clusters of pure *A*. *coluzzii* and *A*. *gambiae* mosquitoes include individuals captured both from larval pools and as indoor-resting adults, while mosquitoes of the Goundry form were found in larval pools but were absent from collections of indoor-resting adults, consistent with their apparently exophilic behavior [14]. Samples were also overlaid with the karyotype of the paracentric 2La inversion (Fig 1C) as determined by a molecular diagnostic assay [33], and the genotype for the nucleotide mutation of the *para* gene associated with pyrethroid insecticide resistance (*kdr*, Fig 1D) [34]. The same four major population groups are detected using half the number of markers (n = 400 randomly chosen SNPs, Fig 2). Similarly, analysis of samples by individual year (i.e., malaria transmission season) yields the same population clusters (Fig 3) with no detectable difference in the relative proportions of the three population groups across the two transmission seasons (chi-square = 0.457, df = 2, p = 0.796). The stability of the PCA results indicates that identification of major subgroups for this local population is comprehensive, and that it is unlikely that other major genome wide subdivision is present in the population sample.

### Genetic association for susceptibility to *P*. *falciparum*

The Goundry subgroup displays significantly higher susceptibility to infection with wild *P*. *falciparum* as compared to *A*. *coluzzii* and *A*. *gambiae* (p<1*10^−4^), consistent with previous observations [14] but here confirmed with independent samples. We also find no difference for *P*. *falciparum* infection susceptibility between *A*. *coluzzii* and *A*. *gambiae* (p = 0.31), which is in accord with multiple published reports [35–39].

### Genomic patterns of LD and recombination within population subgroups

**Fig 4:**
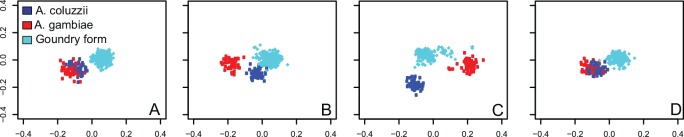
Sliding window PCA indicates that differentiation between *A*. *coluzzii* and *A*. *gambiae* is restricted to centromeric chromosome regions. Chromosome 3 is analyzed as four non-overlapping windows of 115 uniformly spaced SNPs each, as follows: A) Telomeric and central region of chromosome 3R, B) centromeric region of chromosome 3R, C) centromeric region of chromosome 3L, and D) central and telomeric region of chromosome 3L. Sliding window analysis indicates that *A*. *coluzzii* (blue) and *A*. *gambiae* mosquitoes (red) cluster together as an apparently panmictic population when typed using non-centromeric markers. In contrast, the Goundry form (turquoise) is distinct from *A*. *coluzzii* and *A*. *gambiae* across the entire length of both chromosome arms, at both centromeric and non-centromeric sites.

Genome-wide marker density in the current study is substantially higher than the density of microsatellites previously employed in population-level studies using similarly large sample sizes [11, 14], and consequently permits examination of finer patterns of genomic differentiation between taxa. Markers on chromosome 3 have been previously employed as essentially neutral loci to estimate genome-wide differentiation, independent of potentially confounding features such as inversions or major *A*. *coluzzii/gambiae*-related elements such as SI [10, 11, 14, 40]. Non-overlapping sliding window analysis of uniformly spaced SNPs across chromosome 3 indicates that there is little or no differentiation between *A*. *coluzzii* and *A*. *gambiae* across most of the genome (Fig 4A and 4D), consistent with reports of extensive gene flow between them [14, 16, 17, 32, 41]. The greatest levels of differentiation between *A*. *coluzzii* and *A*. *gambiae* are localized in the centromeric SI (Fig 4B and 4C). In distinction, the Goundry group diverges sharply from *A*. *coluzzii* and *A*. *gambiae* across the genome, even in the windows that do not separate *A*. *coluzzii* and *A*. *gambiae* (Fig 4A and 4D).

**Fig 5:**
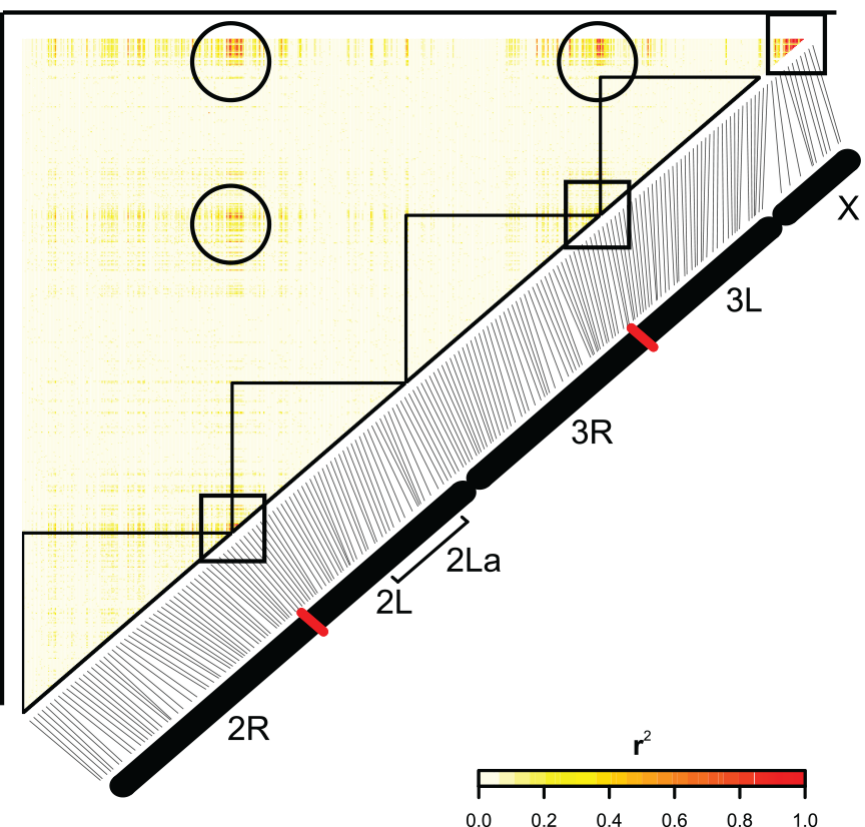
Signals of population differentiation between *A*. *gambiae* and *A*. *coluzzii*. We screened for genome-wide linkage disequilibrium (LD) outside the centromeric Speciation Islands (SI). The individual SNP that is the most informative for the observed genome-wide disequilibrium between *A*. *coluzzii* and *A*. *gambiae* is position X.23852135, located within the X-chromosome SI (see Methods). This SNP was tested for LD with all other genome wide SNPs at an r^2^>0.5, minor allele frequency ≥10%. The plot indicates SNPs highly correlated with X.23852135 under these parameters. 66 SNPs outside of centromeric SI met selection and quality criteria as new candidate markers of subgroup/sister taxa differentiation (S2 Table). Circles highlight linkage patterns across chromosomes, while squares indicate the high-LD centromeric regions of each chromosome.

**Fig 6:**
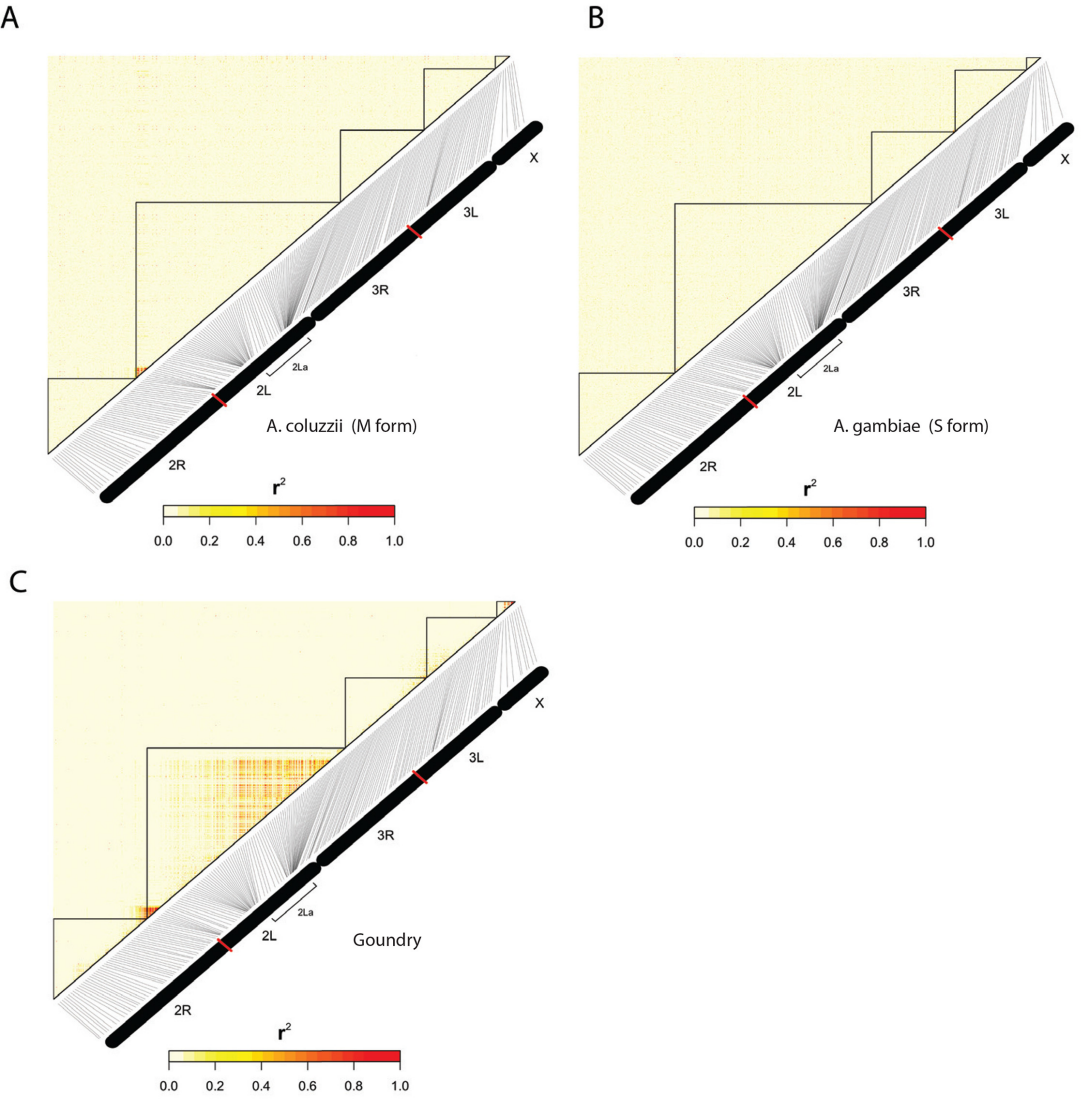
Genome wide linkage disequilibrium within population subgroups. LD was measured by r^2^ for A) *A*. *coluzzii*, B) *A*. *gambiae* and C) the Goundry form. At the study site, the 2La inversion is nearly fixed in *A*. *coluzzii* and *A*. *gambiae* but segregates in the Goundry form, hence the detectable LD across the 2La inversion only in Goundry. Also, the centromeric region of the second chromosome carrying the insecticide resistance mutation, kdr of the para gene [44] is largely fixed in *A*. *gambiae* but segregates in both *A*. *coluzzii* and Goundry forms. These plots include all SNPs that passed quality control and were not fixed within population taxa.

We scanned the genomes of the *A*. *coluzzii* and *A*. *gambiae* for signals of population genetic differentiation, in order to identify positions displaying long-range LD beyond the well-studied SI of the centromeric regions. Local correlation due to physical linkage on the chromosome is evident across centromeric regions (Fig 5, boxes), consistent with the low recombination rates in centromeres. Marked linkage disequilibrium is also detected across chromosomes between physically unlinked sites (Fig 5, circles), consistent with locations of the centromeric SI [42]. Because the X-chromosome SI is the main driver of the observed genome-wide disequilibrium between *A*. *coluzzii* and *A*. *gambiae* ([28] and Fig 5) we screened for genome-wide SNPs that display high r^2^ values with the subgroup-diagnostic X-chromosome SI. Measuring LD of genome-wide SNPs with positions highly diagnostic for the underlying population subdivision should be more informative than simple genome-wide F_ST_ measurement. We identified 66 SNPs that met the selection and quality criteria (Fig 5 and S2 Table). The candidates are distributed over 45 genes, thus some genes carry multiple SNPs. Of these, 24 SNPs in 20 genes lie outside the previously identified centromeric SIs. Only one of these genes (*Tep3*) has been previously implicated in *A*. *gambiae*/*A*. *coluzzii* differentiation [43], and thus the other 19 represent novel candidate genes associated with population differentiation between the two species. Known or predicted gene functional categories include immunity, nervous system and development (S2 Table), and offer multiple plausible candidates for follow-up studies, including testing within *A*. *coluzzii* and *A gambiae* populations at other sites where they are sympatric. In contrast to the above among-subgroup analysis, LD signals within population subgroups appeared as expected for the SNP marker density, detectable mainly at centromeres and segregating inversions (Fig 6).

### Candidate diagnostic SNPs for molecular attributes

We identified a set of 21 candidate SNPs that were highly informative for the detection of mosquito genetic attributes. Seven highly informative SNPs were identified for each attribute, i) karyotype of the 2La inversion, ii) genotype of the *para* gene *kdr* mutation associated with pyrethroid resistance, and iii) *A*. *gambiae*/*A*. *coluzzii* differentiation. Sequenom genotyping assays were developed and 80 individual samples were genotyped (S3 Table). Genotype calls from Illumina and Sequenom were highly concordant. The SNPs represent a candidate diagnostic set highly efficient for the local population in Burkina Faso, but as yet untested for samples from other geographic sites. Diagnostic utility of these candidate SNPs for the research community will thus require additional confirmation in other populations.

## Discussion

### Population structure determined by local population sampling

We sampled a local West African mosquito population over time and genotyped it with a large number of genome-wide markers selected for information content, but without regard to gene functional category. This approach yielded a comprehensive characterization of local population substructure, an important prerequisite for accurate assessment of vector control interventions, as well as for association studies linking measured phenotype to underlying genotype. The use of 800 markers in a ~280 Mb genome was more than sufficient to detect the level of population subdivision that, if left undetected, would likely lead to spurious results in a genome wide association study [45]. As few as 400 random markers (~2 markers/Mb) were adequate to detect the same major subdivisions.

Although whole-genome resequencing has become more accessible, nevertheless the analysis of >400 mosquitoes from one geographic site by resequencing for a single project would be costly. The SNP genotyping results obtained here have been used to identify small numbers of candidate ancestry-informative SNPs for different attributes (S3 Table). However, general applicability of this SNP set for other mosquito populations will require additional validation using samples collected over the species/attribute range. In the end, simplified, ideally field deployable assays allow routine acquisition of deep population genetic information from large-scale field surveys done for biological studies or evaluation of vector control.

Regarding the Goundry form, the desirable SNPs for a diagnostic assay would be the fixed differences present in Goundry and absent from non-Goundry individuals. SNPs identified from the current study were ascertained from available *A*. *gambiae* and *A*. *coluzzii* genome sequence. Some of these variants display under or over enrichment in Goundry and can be used for a partially-efficient probabilistic assay, but by definition the Goundry fixed differences that would be most informative tool cannot be identified from non-Goundry sequence, and must await whole genome sequences from Goundry mosquitoes.

### New candidate loci for population differentiation between *A*. *coluzzii* and *A*. *gambiae*

The mechanisms of mating isolation and assortative mating between *A*. *coluzzii* and *A*. *gambiae* are not known, but appear to be largely prezygotic because the species hybridize in the laboratory [46, 47]. The known genomic regions of highest genetic differentiation between *A*. *coluzzii* and *A*. *gambiae* are the SI in the centromeres [17, 32], but this likely stems from ascertainment bias because previous studies used minimal marker density and/or sample depth, and under those conditions the power to detect differentiation is largely limited to regions of extended LD, such as centromeres. It is also likely that centromeric regions will retain a historic signal of differentiation longer due to the diminished rates of recombination. We now find 24 SNPs in 20 genes outside of the centromeric regions that highly correlate with the X chromosome diagnostic for *A*. *coluzzii* and *A*. *gambiae*. None of these SNPs occur in the 2R non-centromeric island published by Turner et al. [17]. Five of these SNPs occur in a single gene, *Tep3*, and a 100kb genomic region containing *Tep3* was previously highlighted as differentiated between *A*. *gambiae* and *A*. *coluzzii* by White et al. [43]. Thus, we report previously unrecognized cases of 19 genes that contain a significantly differentiated SNP and represent new candidate loci for association with population differentiation phenomena such as reproductive isolation and subgroup-specific adaptation between *A*. *coluzzii* and *A*. *gambiae* mosquitoes.

Of the 19 newly-identified non-centromeric genes (S2 Table), one has predicted function in wing imaginal disc development. There are reported differences in wing morphology between *A*. *coluzzii* and *A*. *gambiae* mosquitoes that are proposed to underlie the production of different wingbeat harmonic frequencies, thus permitting mate discrimination by *A*. *coluzzii* and *A*. *gambiae* mosquitoes [48, 49]. Two new candidates have established roles in immunity (Toll1A, SRPN4 [50–53]), along with Tep 3. These immune genes could be associated with the previously hypothesized exposure of the population subgroups to distinct pathogen profiles in different ecological habitats [43, 54, 55]. Finally, four other candidates with predicted central nervous system functions could underlie observed behavioral differences tied to ecological specialization between *A*. *coluzzii* and *A*. *gambiae* for oviposition site choice, formation of mating swarms, or other phenotypes [21, 23, 56]. The twelve other candidate genes have little functional data. Together, these genes represent new candidate loci located outside the previously-studied centromeric SI intervals, potentially associated with features of population differentiation between *A*. *coluzzii* and *A*. *gambiae*. Because we analyzed sympatric mosquitoes collected from a single defined geographic region, geographic variables do not underlie the differentiation signal, although the results cannot necessarily be generalized to populations in other regions of West Africa without sampling and testing at other sympatric sites.

## Materials and Methods

### Mosquito sampling and *P*. *falciparum* infection

Mosquitoes were sampled as larvae using the standard dipping method or as adults by aspirator catch, as previously described in detail [14]. Mosquitoes were collected in the Sudan Savanna region of Burkina Faso in the village of Goundry (12°30´N, 1°20´W), 30 km N of the capital city, Ouagadougou, across months of the rainy season during the 2007 and 2008 malaria transmission seasons [57]. Permission was obtained from Goundry village authorities to collect mosquitoes in the village. Larval-caught *A*. *gambiae* species complex mosquitoes were brought to the insectary in Ouagadougou where they were raised under standard laboratory rearing conditions to adulthood. Following emergence, 3 day old adults were challenged with wild *P*. *falciparum* by experimental infection. Feeding was done on an artificial membrane in a water-jacketed feeding device as described previously using gametocytemic blood obtained from study participants [35]. Unfed mosquitoes were excluded from analysis and infection levels for fed mosquitoes were determined by counting midgut oocysts 7–8 days post infection. Genomic DNA was extracted from carcasses for genotyping.

### Illumina chip design and hybridization

To design the custom SNP chip, polymorphism data were combined from individual sources [54, 55, 58] as well as an analysis of the *A*. *coluzzii* and *A*. *gambiae* genome sequences available at Vector Base. At the time of the chip design, the *A*. *coluzzii* and *A*. *gambiae* genome assemblies were not available at VectorBase and raw sequence read data was used for SNP design. SNPs were identified by alignment of the *A*. *coluzzii* and *A*. *gambiae* sequence reads against the assembled genome of the PEST strain using BLAST. We summarized all high confidence alignments in a simple frequency table. For every position in the PEST genome we recorded the number of A,G,C,T nucleotides observed for that position. To be considered viable for inclusion on the chip, a SNP had to meet the following criteria: i) have a minimum read depth of 10, ii) be surrounded by ~200 bp of SNP free-sequence, iii) be variable across any set of samples used for SNP ascertainment, iv) have a minor allele frequency of at least 15%. We submitted to Illumina 5995 candidates, 4840 from shotgun sequence and 1155 from 3 independent deep re-sequencing projects. The final catalog of 1536 SNPs was selected from 3394 SNPs that passed Illumina design criteria, 1358 from shotgun sequence and 178 from deep sequencing projects. The complete set of SNPs typed on the Illumina chip and their primers is available in S1 Table. The chip includes a uniformly-spaced genome-wide marker set (n = 812), as well as additional marker coverage (n = 724) within certain genomic features such as chromosomal Speciation Islands (SI). Overall, the chip types 1536 SNPs, with an average density of 1 marker every ~340 kb for the uniformly-spaced set. The chip is thus well-powered for accurate and comprehensive detection of population stratification and related genome features, although not for genome-wide association given that linkage disequilibrium (LD) in *A*. *gambiae* decays to uninformative levels on average within <500 bp [54]. Hybridization of the chips was done using standard Illumina procedures in the Boston Children’s Hospital Molecular Genetics Core Facility (IDDRC).

### Genotyping and data analysis

Due to the low quantity of DNA available from individual mosquitoes, all DNA samples were subjected to whole genome amplification (Genomiphi, GE Health Sciences) using supplied protocols. DNA was then ethanol precipitated, concentrations determined by the Picogreen method [59] and 500 ng submittted for Illumina chip hybridization. We used a two stage approach, hybridizing a pilot (n = 96) and an expanded (n = 384) set of samples. The first 96 samples were used to validate array performance and included duplicates (n = 24) to verify reproducibility of genotype calls and provide quality control metrics. All mosquitoes genotyped in the larger expanded set of samples came from five successful experimental infections as defined previously [4, 60], briefly, sessions with oocyst infection prevalence ≥30% and oocyst intensity in at least one individual mosquito in the infected group of ≥10 oocysts. This infection quality-control cutoff assures that all analyzed individuals were exposed to an experimental infection with the power to distinguish levels of susceptibility, free from confounding technical or other factors influencing infection success. Of the 456 unique samples genotyped here, only 160 samples (35%) were previously genotyped and analyzed, using <10 microsatellites on chromosome 3 [14]. Thus, genotyping in the current study was carried out at much higher marker density than in the previous study.

Data were analyzed using the BeadStudio package (Illumina) following the manufacturer's guidelines [61]. Quality control was carried out in two steps: i) Manual curation. Following standards recommended by the manufacturer, boundaries of poorly clustered SNPs were either manually redefined or the SNPs were removed. Because we expected distinct population subgroups segregating within our overall sample, we used Hardy Weinberg Equilibrium (HWE) statistics as a trigger for manual inspection but we did not reject well-clustered SNPs violating HWE. In addition, samples with low call rate were removed, which left more than 88% of samples showing a call rate higher than 85%. ii) SNP call rate. SNPs were removed if they failed in more than 25% of the mosquitoes, which resulted in removal from the analysis of only 89 SNPs (~6%). After application of all QC filters, high-quality data remained for 422 mosquito samples for 1447 genome-wide SNPs, yielding a 94% SNP conversion rate. These 422 samples included 56 A. coluzzii, 52 A. gambiae, 284 Goundry form, and 30 A. arabiensis. The distribution of GenTrain scores, a metric of genotype quality for GoldenGate assays (produced by an algorithm implemented in the Illumina software application, BeadArray GenCall [61]) is shown for SNPs passing the above QC filters (S1 Fig). For PCA analyses presented in Figs 1–4, standard multidimensional scaling as implemented in R (cmdscale in the Stats package) was used for clustering.

A subset of samples (n = 24) were hybridized in duplicate, and over 99% of called genotypes were concordant. For additional validation of genotype calls using an independent technology and to test a set of SNPs with high informative value for molecular attributes, a subset of 21 SNPs were converted to Sequenom assays and 80 mosquito samples genotyped by this independent method. Across all 21 SNPs, the genotype concordance between Illumina and Sequenom averaged 95.5%, ranging from 89% to 99% (S2 Fig). Sequenom Mass Array genotyping was done at the University of Minnesota Genomics Center.

### Analysis of infection phenotypes

To test for differences in infection susceptibility across subgroups, analyses were carried out with infection as a blocking factor, and p-values were determined for each individual infection using the Chi Square test and combined p-values across infections via the method of R.A. Fisher [62]. Most of the individuals in the expanded sample set (n = 335) had accompanying infection phenotype data. The phenotyped sample set of 335 were generated from five independent experimental infections, with each infection averaging 67 individuals (range 39–89 individuals). Each experimental infection included individuals from each of the 3 population groups, *A*. *gambiae*, *A*. *coluzzii* and the Goundry form.

### Population subgroup differentiation and detection of differentiated SNPs

Linkage disequilibrium (LD), as analyzed and depicted in Figs 5 and 6, was computed using the LD() function from the genetics package in the R statistical package. For plotting the LD map, the image() function was used. The scale bar was drawn with the function image.plot() from within the fields package in R.

To identify SNP genetic correlation across chromosomes as shown in the centromeric regions (boxes in Fig 5), a selection filter was applied to all *A*. *coluzzii* and *A*. *gambiae* mosquitoes. Centromeric regions were defined as +/-5Mb from the centromere for a total area of 10Mb, 5Mb on each chromosome arm. Initially, we determined the individual SNP that was in LD (r2>0.8) with the maximum number of other SNPs across the genome, imposing a SNP inclusion cutoff at minor allele frequency ≥10%. This SNP was on the X chromosome at position 23852135. This region of the X chromosome is the most informative for assignment of *A*. *coluzzii* and *A*. *gambiae* [28]. This SNP was then used in a second screen to find all other genome wide SNPs in LD with this SNP (X.23852135) at an r^2^>0.5, minor allele frequency ≥10%. These SNPs, each individually highly correlated with the X.23852135, are presented in S2 Table. The 66 SNPs that mark differentiation outside speciation islands were specifically quality-controlled by examining the distribution of their GenTrain scores, and there was no difference between the distribution of these 66 markers and the rest of the markers that passed controls (Wilcoxon rank test p = 0.26 and S1 Fig).

## Ethical considerations

For collection of blood from *P*. *falciparum* gametocyte carriers for experimental membrane feeder infection of mosquitoes, the study protocol was reviewed and approved by the national health ethical review board IRB (Commission Nationale d’Ethique en Santé) of Burkina Faso, which issued ethical protocol N° 2006–032 for the described studies. The study procedures, benefits and risks were explained to subjects and their written informed consent was obtained. The consent procedure was approved by the IRB. Subjects who had given consent were brought to CNRFP the day of the experiment for gametocyte carrier screening. All children were followed and symptomatic subjects were treated with the combination of artemether-lumefantrine (Coartem) according to relevant regulations of the Burkina Faso Ministry of Health.

## Supporting Information

S1 Fig Distribution of GenTrain scores for SNPs passing QC filters and used in subsequent analyses. SNPs indicated in red are the set of 66 that show greatest differentiation between *A*. *coluzzii* and *A*. *gambiae* (see S2 Table).
(EPS)

S2 Fig High concordance of genotype calls for SNPs typed by Illumina chip and Sequenom mass array. Twenty-one SNPs were typed on a set of 80 individual mosquito samples. Individual SNPs are shown on the x-axis, and concordance rates between genotype calls from the two distinct technologies are indicated on the y axis.
(EPS)

S1 Table Catalogue of polymorphic SNPs typed by Illumina Golden Gate Assays. (XLSX)

S2 Table Catalogue of genome wide SNPs displaying maximum r2 with the X chromosome speciation island SNP most diagnostic for differentiation of A. coluzzii and A. gambiae. (DOCX)

S3 Table SNPs derived from Illumina chip data with high informative value for detection of mosquito attributes. (DOCX)

S4 Table Genotype data for mosquito individuals typed on the Illumina chip. (XLSX)
