# Supplementary material for: Genetic Structure of a Local Population of the Anopheles gambiae Complex in Burkina Faso
Source: PLoS One. 2016 Jan 5;11(1):e0145308. doi: 10.1371/journal.pone.0145308 (PMC4701492; doi:10.1371/journal.pone.0145308)
Supplement: S2 Table — (DOCX) [file pone.0145308.s004.docx]

| Supplementary Table S2. Catalogue of genome wide SNPs displaying maximum r2 with the X chromosome speciation island SNP most diagnostic for differentiation of A. coluzzii and A. gambiae. | | | | | | | | | | | |
| --- | --- | --- | --- | --- | --- | --- | --- | --- | --- | --- | --- |
|  |  |  |  |  |  |  |  |  |  |  |  |
| **Chromosome** | **Chromosome position (bp)** | **r**^2 a^ | **GeneID** | **Gene/Protein Identity if known** | **Centromeric (Y/N)**^b^ | **Gene Ontology Description** | **GenTrain Scores**^c^ |  |  |  |  |
| 2R | 7873329 | 0.67 | AGAP001674 |  | N | neurogenesis | 0.822 |  |  |  |  |
| 2R | 36368990 | 0.57 | AGAP003349 |  | N |  | 0.566 |  |  |  |  |
| 2R | 39448568 | 0.56 | AGAP003551 |  | N |  | 0.722 |  |  |  |  |
| 2R | 49356895 | 0.54 | AGAP004066 |  | N |  | 0.578 |  |  |  |  |
| 2R | 52461796 | 0.57 | AGAP004229 |  | N |  | 0.731 |  |  |  |  |
| 2R | 60779748 | 0.68 | AGAP004667 |  | Y |  | 0.543 |  |  |  |  |
| 2R | 61305464 | 0.81 | AGAP004674 |  | Y |  | 0.923 |  |  |  |  |
| 2R | 61415220 | 0.83 | AGAP004675 |  | Y |  | 0.760 |  |  |  |  |
| 2R | 61415742 | 0.83 | AGAP004675 |  | Y |  | 0.583 |  |  |  |  |
| 2L | 746341 | 0.59 | AGAP004686 |  | Y |  | 0.785 |  |  |  |  |
| 2L | 1272741 | 0.70 | AGAP004691 |  | Y | wing imaginal disc dev. neurogenesis | 0.766 |  |  |  |  |
| 2L | 1414825 | 0.66 | AGAP004692 |  | Y | wing imaginal disc dev. | 0.785 |  |  |  |  |
| 2L | 1834405 | 0.72 | AGAP004695 |  | Y |  | 0.780 |  |  |  |  |
| 2L | 1834476 | 0.70 | AGAP004695 |  | Y |  | 0.674 |  |  |  |  |
| 2L | 1835085 | 0.69 | AGAP004695 |  | Y |  | 0.591 |  |  |  |  |
| 2L | 1970368 | 0.73 | AGAP004698 |  | Y |  | 0.764 |  |  |  |  |
| 2L | 1976160 | 0.55 | AGAP004699 |  | Y |  | 0.714 |  |  |  |  |
| 2L | 2081228 | 0.72 | AGAP004701 |  | Y |  | 0.796 |  |  |  |  |
| 2L | 2081554 | 0.73 | AGAP004701 |  | Y |  | 0.670 |  |  |  |  |
| 2L | 2119508 | 0.70 | AGAP004701 |  | Y |  | 0.772 |  |  |  |  |
| 2L | 2252367 | 0.77 | AGAP004703 |  | Y |  | 0.845 |  |  |  |  |
| 2L | 2252967 | 0.59 | AGAP004703 |  | Y |  | 0.621 |  |  |  |  |
| 2L | 2400065 | 0.62 | AGAP004707 | para voltage gated sodium ion channel gene kdr | Y |  | 0.704 |  |  |  |  |
| 2L | 2425016 | 0.66 | AGAP004707 | para voltage gated sodium ion channel gene kdr | Y |  | 0.728 |  |  |  |  |
| 2L | 2430363 | 0.71 | AGAP004707 | para voltage gated sodium ion channel gene kdr | Y |  | 0.491 |  |  |  |  |
| 2L | 2430786 | 0.67 | AGAP004707 | para voltage gated sodium ion channel gene kdr | Y |  | 0.770 |  |  |  |  |
| 2L | 2431194 | 0.66 | AGAP004707 | para voltage gated sodium ion channel gene kdr | Y |  | 0.497 |  |  |  |  |
| 2L | 2472772 | 0.73 | AGAP004708 | arginine-tRNA synthase | Y |  | 0.767 |  |  |  |  |
| 2L | 2473048 | 0.69 | AGAP004708 | arginine-tRNA synthase | Y |  | 0.786 |  |  |  |  |
| 2L | 2489023 | 0.67 | AGAP004711 |  | Y |  | 0.783 |  |  |  |  |
| 2L | 2489212 | 0.69 | AGAP004711 |  | Y |  | 0.784 |  |  |  |  |
| 2L | 5862839 | 0.53 | AGAP004914 |  | N |  | 0.718 |  |  |  |  |
| 2L | 11163943 | 0.52 | AGAP005165 |  | N | neurogenesis | 0.795 |  |  |  |  |
| 2L | 34857444 | 0.69 | AGAP006632 |  | N |  | 0.506 |  |  |  |  |
| 3R | 177063 | 0.51 | AGAP007742 |  | N |  | 0.518 |  |  |  |  |
| 3R | 38150974 | 0.59 | AGAP009670 | SRPN4 | N | immune response | 0.505 |  |  |  |  |
| 3R | 43669475 | 0.53 | AGAP009803 | GPRGR11 | N | sensory perception of taste | 0.503 |  |  |  |  |
| 3R | 45614927 | 0.78 | AGAP009934 |  | N |  | 0.532 |  |  |  |  |
| 3R | 53005331 | 0.76 | AGAP010305 |  | Y | neurogenesis | 0.482 |  |  |  |  |
| 3R | 53006186 | 0.76 | AGAP010305 |  | Y | neurogenesis | 0.490 |  |  |  |  |
| 3R | 53091591 | 0.84 | AGAP010308 |  | Y |  | 0.535 |  |  |  |  |
| 3R | 53091720 | 0.90 | AGAP010308 |  | Y |  | 0.602 |  |  |  |  |
| 3L | 304874 | 0.88 | AGAP010313 |  | Y | oogenesis | 0.748 |  |  |  |  |
| 3L | 357584 | 0.90 | AGAP010314 |  | Y |  | 0.805 |  |  |  |  |
| 3L | 398426 | 0.92 | AGAP010316 |  | Y |  | 0.773 |  |  |  |  |
| 3L | 10279185 | 0.62 | AGAP010781 |  | N |  | 0.753 |  |  |  |  |
| 3L | 11216951 | 0.90 | AGAP010816 | TEP3 | N | immune response | 0.592 |  |  |  |  |
| 3L | 11217837 | 0.93 | AGAP010816 | TEP3 | N | immune response | 0.783 |  |  |  |  |
| 3L | 11217945 | 0.79 | AGAP010816 | TEP3 | N | immune response | 0.697 |  |  |  |  |
| 3L | 11218225 | 0.92 | AGAP010816 | TEP3 | N | immune response | 0.569 |  |  |  |  |
| 3L | 11220075 | 0.63 | AGAP010816 | TEP3 | N | immune response | 0.492 |  |  |  |  |
| X | 15360416 | 0.70 | AGAP000829 |  | N |  | 0.770 |  |  |  |  |
| X | 16620585 | 0.55 | AGAP000877 | Cytochrome P450 | N |  | 0.625 |  |  |  |  |
| X | 17513955 | 0.68 | AGAP000929 |  | N | wing imaginal disc dev. | 0.703 |  |  |  |  |
| X | 18742681 | 0.94 | AGAP000974 |  | N |  | 0.747 |  |  |  |  |
| X | 18817514 | 0.98 | AGAP000981 |  | N |  | 0.525 |  |  |  |  |
| X | 19060971 | 0.80 | AGAP001004 | Toll1A | N | neurogenesis immune response | 0.521 |  |  |  |  |
| X | 19834390 | 0.92 | AGAP001034 |  | Y |  | 0.474 |  |  |  |  |
| X | 20061764 | 0.92 | AGAP001041 |  | Y |  | 0.736 |  |  |  |  |
| X | 20061827 | 0.98 | AGAP001041 |  | Y |  | 0.780 |  |  |  |  |
| X | 20063990 | 0.98 | AGAP001042 |  | Y |  | 0.749 |  |  |  |  |
| X | 20955148 | 0.96 | AGAP001050 |  | Y |  | 0.693 |  |  |  |  |
| X | 22105305 | 0.98 | AGAP001061 |  | Y | wing imaginal disc dev. | 0.667 |  |  |  |  |
| X | 23467702 | 0.98 | AGAP001082 |  | Y |  | 0.580 |  |  |  |  |
| X | 23478195 | 0.83 | AGAP001082 |  | Y |  | 0.608 |  |  |  |  |
| X | 23512358 | 0.96 | AGAP001084 |  | Y |  | 0.559 |  |  |  |  |
|  |  |  |  |  |  |  |  |  |  |  |  |
|  |  |  |  |  |  |  |  |  |  |  |  |
| ^a^ r^2^ between the given SNP and the SNP at X.23852135 | | |  |  |  |  |  |  |  |  |  |
| ^b^ Centromeric was defined as ± 5MB from the centromeric end of the chromosome arm | | | | |  |  |  |  |  |  |  |
| ^c^ GenTrain scores, as defined by Illumina, are a measure of genotype quality and values above 0.40 are considered good. | | | | | |  |  |  |  |  |  |
