# Supplementary material for: Genetic Structure of a Local Population of the Anopheles gambiae Complex in Burkina Faso
Source: PLoS One. 2016 Jan 5;11(1):e0145308. doi: 10.1371/journal.pone.0145308 (PMC4701492; doi:10.1371/journal.pone.0145308)
Supplement: S3 Table — (DOCX) [file pone.0145308.s005.docx]

| **For *A. gambiae* vs *A. coluzzii*** | **Chr** | **Coordinate** | **Concordance rates**** |
| --- | --- | --- | --- |
| CGCAGCAGACCCATTTCTGATTCTGAGCCGTATGAACTAATATTATCGAT[T/C]GACATGGCCCTTTGAGTGATCCGGGTGCGCCATGAATCTGATCGGCGCTG | X | 22105305 | 90 |
| GCTGCATCCGTCGCAGCATCACAACAGGCCCAACCGCAAGAGGAAGAGCG[C/G]TACTGCTGGGTGTGCTTTGCGACCGAGGAGGATGATAAGGTAGCGCCGTG | X | 20061827 | 95 |
| CAATCAAATGGAAGTCGCGATAGATTTTGTAACCTGCAATGTTATAACTC[A/G]TAATATCGACACCTTGCCACGATTGATGACAGGTTTTAATTTTAGCCGAA | X | 20955148 | 96.2 |
| TTTCTTATTTTCAGACTAAAAATTTCAAATACACACACCGAGAGTCTTGA[A/G]ACGCCGACACCGACTAGCAGTGGCTTCATTCCCCTCGTAGGCGTTAGTTT | X | 24141427 | 98.8 |
| CCTGCATTTCGGTGTCGGTTAAGCTAAAGCGTTCCAACAGCTGAGTTTTC[T/C]TGGATGCTAGAAGGTAGAACAGCACCATGGCGAACGCCACGATCACCATG | X | 20063990 | 98.8 |
| TGGGTTTTTTGCCCAATACGTACGTTTTCACCAAAAGTTTAGCGGAACAT[G/A]TTGTCAACGAGCATAAGGATCGCTTACCGATAATTTTGTTTCGTCCATCA | X | 23512358 | 92.4 |
| TTTTGAATGGAATGCTTCAGCTGGTACATCATATCTATACCATACATAGT[A/G]AACAGCTTCTCACATAGCGTGAAGTAATCTCCCTGAAGTGAATTACAAAC | X | 23467702 | 96.2 |
| **For 2La inversion karyotype** |  |  |  |
| ATCCAGAACTCGCCGGCCGGTGTGCCGAACCCTTGGGCGTACTCTTCCCA[C/G]GAGCGGTTAAAGTCAACCGTACCGTCCTGGCGCCGCTGCACCGTCGTCCA | 2L | 41165721 | 97.5 |
| AATGTGTTGGCCCTCAGATGGGAAAGCTTTAAGATTAAAGTATCGTTCGG[G/A]ATATGGGCAAGCAAAAATGTGCCTTCCTCGCTGGGAGCCCACTTCTTTAT | 2L | 41061736 | 91.2 |
| GCGGCCATTCGCAAGATGTTCACGCTGTACACGACCGATCCGCTGAATGA[A/G]CATCGCGACATTAACGATGTGTGGCGCAAATTTATGGCACTGTTCATCTG | 2L | 39857195 | 97.5 |
| CTCGCCTGGCAGAGCTTAAACTACTCAACGTGAGCTGGAATCGGCTCACA[T/G]CATTCGAAAGCATAATCCTGCCCCCGGAAAACCAACTCCAAGTGCTCGAT | 2L | 41649500 | 96.2 |
| TATACACACATGCTCAAACAGAACTTAGATACCTAACGTATACTTGTAAT[A/G]CAGTAGAAATTCAAATGAAGTAGCCGTTAATAGCCGTTTTTCGTATTGTA | 2L | 40189213 | 96.2 |
| TGAAAGTACGGATTGGTCAGCAGGCAGCTTTCGTCGTAGTACTCGCTCAG[T/C]GCCGGATAGGACGCGATGATCGGTACGGTCGCCTTCCGCCAGAACCGCAT | 2L | 40178476 | 91.1 |
| CGTCTGCAAAGCCTATCACTCGGCAGTAACCTCATCCACCAGCTACACCC[T/C]CAGTCGTTCGCTCGCCTGGCAGAGCTTAAACTACTCAACGTGAGCTGGAA | 2L | 41649439 | 98.8 |
| **For the *kdr* insecticide resistance mutation** |  |  |  |
| ACACGATATTTGTGTCAATCCAAATCAGAAGGGTTACCTGGTTACATAGT[A/G]GAGACACTAAAAATAATAAACGAAAAGGAAATTATTCCAGAGGGATTTTC | 2L | 1834476 | 97.5 |
| TGTACTTCCGCCGCCTCCTCGATCCACCTGCCATGAAGTAGGTCCTCCGC[T/C]CATTGGACCTCCATCATTGTGGCCGCCTCCACCTCCGCTGCCGCCGCTAA | 2L | 1272741 | 96.2 |
| GCTCGATCAGTACGTGTGGGCCAAGCAAACCAACAGCAGGTACAACCAAT[T/C]CCTCAGGACGAACGCCGTTTACAATTACCAAGCGATTGACGCGAATGGGC | 2L | 2081228 | 95 |
| ACTCAAGACGTGGAATACGTAAAGCAGGAGGCAAAAGTTGTGTATTTGTT[G/A]GAGTGTTTGCAGAAAACACCACCTCCAGTACTGATATTTGCTGAGAAAAA | 2L | 2489023 | 98.8 |
| TATCGGTCGGTCGAGTCATTTAGAAATCAAGAGAAGGACGTGCTAGTTGC[T/C]ACTGACGTCGCATCTAAAGGTTTGGATTTTCCGGATGTGCAGCATGTAAT | 2L | 2489212 | 94.9 |
| CACATTCCTGTCTGACCCGCGGTTTTGCGAGAGCCTCTTTCCCATGGTTC[C/T]AAGTGTTTCACTTGATAGTATATTTCGTCTACAACTTCATGGTAAGTCTT | 2L | 1970368 | 88.8 |
| TATCTTGTAATAAGTTTCCTTATCGTTATTAACATGTACATTGCTGTTAT[C/T]CTCGAAAACTACTCGCAAGCTACGGAAGATGTTCAAGAAGGCTTAACTGA | 2L | 2430786 | 98.8 |
|  |  |  |  |
|  |  |  |  |
| * The SNPs are given in brackets with flanking genomic sequence (+/- 50bp) |  |  |  |
| ** Concordance in genotype for 80 individuals typed by both Illumina SNP chip and Sequenom. |  |  |  |

Supplementary Table S3. SNPs derived from Illumina chip data with high informative value for mosquito attributes*
